# Recovering the Magnetic Image of Mars from Satellite Observations

**DOI:** 10.3390/jimaging7110234

**Published:** 2021-11-09

**Authors:** Igor Kolotov, Dmitry Lukyanenko, Inna Stepanova, Yanfei Wang, Anatoly Yagola

**Affiliations:** 1 Department of Mathematics, Faculty of Physics, Lomonosov Moscow State University, 119991 Moscow, Russia; ii.kolotov@physics.msu.ru (I.K.); lukyanenko@physics.msu.ru (D.L.); 2 Moscow Center for Fundamental and Applied Mathematics, 119234 Moscow, Russia; 3 Schmidt Insitute of Physics of Earth, Russian Academy of Sciences, 123995 Moscow, Russia; tet@ifz.ru; 4 Innovation Academy for Earth Science, Chinese Academy of Sciences, Beijing 100029, China; yfwang@mail.iggcas.ac.cn; 5 Key Laboratory of Petroleum Resources Research, Institute of Geology and Geophysics, Chinese Academy of Sciences, Beijing 100029, China; 6 University of the Chinese Academy of Sciences, Beijing 100049, China

**Keywords:** inverse problem, magnetic parameters reconstruction, full tensor magnetic gradient, regularization, 45Q05, 65Rxx, 85A99, 86A22

## Abstract

One of the possible approaches to reconstructing the map of the distribution of magnetization parameters in the crust of Mars from the data of the Mars MAVEN orbiter mission is considered. Possible ways of increasing the accuracy of reconstruction of the magnetic image of Mars are discussed.

## 1. Introduction

The study of the magnetic fields of the planets is one of the ways to obtain information about the internal structure of the planet and its evolution. Access to information about the magnetic fields of planets became possible due to the emergence and development of interplanetary missions. The first results of measuring the magnetic field of Mars were obtained in 1965 by the US mission Mariner 4, which discovered the absence of the global magnetosphere of Mars. In the 1970s, Soviet Mars 2, 3, 5 and later Phobos 2 detected a rather weak magnetic field of about 60 nT in the vicinity of the equator and 120 nT in the vicinity of the pole [1]. In 1996, the MGS (Mars Global Surveyor) mission with a magnetometer reflectometer onboard was sent to Mars [2]. Thanks to this device, data were obtained on the magnetic field of Mars at various altitudes above the surface of Mars. These data made it possible to solve inverse problems of restoration of such physical parameters as magnetization [3,4]. It was found that the crust of Mars is quite strongly magnetized in some areas. From this it was concluded that, although Mars now does not have a global magnetic field, it may have had an active magnetic dynamo earlier [5]. In this regard, modeling of the remanent magnetic field is an important task for studying the deep structure of Mars and for testing models of the magnetic dynamo of Mars in the past, as stated in the literature, see [6,7,8,9,10,11,12,13,14,15]. In the latter case, this allows the creators of magnetic dynamo models to verify their models and examine what happens after it disappears, —whether the distribution of remanent magnetization predicted by these models in the crust of Mars coincides with the values observed today. The experimental data of the MGS mission made it possible to obtain a primary estimate of the global distribution of magnetic field sources in the planet’s crust [6,16].

The most recent research is based on experimental observation data [17] of the MAVEN mission (NASA’s Mars MAVEN orbiter [18] that began work in 2014). A variety of methods developed for studying the Earth’s magnetic field can be applied to studying the magnetic field of Mars (see, for example, [3,4]). Previously widespread approaches to modeling local (the intensely magnetized southern highlands with strong magnetic anomalies reaching 1500 nT at 200 km altitude) [7,8,9] and global regions magnetic field distributions induced by remanent magnetism of the Martian crust were divided into the “spherical harmonic expansions” models [10,11] and the “equivalent source” models [12,13]. Current crustal magnetic field models [14,15] consider the MAVEN magnetometer dataset at an altitude of about 135 km [2]. This is due to the fact that the data obtained by the abovementioned MGS magnetometer are mainly distributed in an orbit with an altitude of 370–430 km, some data are available at an altitude of 90–170 km. Therefore, in these studies, only a part of the data from the MAVEN mission was used in order to compare the results obtained on the basis of the data of the MAVEN magnetometer with the results obtained earlier on the basis of the data of the MGS magnetometer.

The structure of this work is as follows. In Section 2, we describe a model based on restoring magnetization parameters from traditional magnetic data that are total magnetic intensity (TMI) [3,4]. Further, in Section 3 we use this model to reconstruct the equivalent distribution of magnetization parameters in the near-surface layer (crust) of Mars. This approach is similar to the approach proposed in [19]—the method of “sweeping” the sources of fields from a multidimensional region of space to its boundary. On the example of studying the gravitational field of Mars, it was shown [20,21] that the distribution of two-dimensional sources on a plane under the surface of Mars resembles the field itself in outline. In [20], a simulation of anomalous gravity masses distribution in the sourthwestern part of Elysium Planitia under the surface was performed on the base of the modified S-approximation method. The same technique was used to restore the equivalent magnetic sources in [22]. The results obtained in these works are based on the so-called linear integral representation method proposed in [23,24] and then evolved in [25,26,27,28,29,30]. Therefore, the approach we are considering to restore the equivalent distribution of magnetic field sources is encouraging. The proposed algorithm was used to process the magnetic data of the MAVEN mission at all altitudes. The obtained results demonstrate the effectiveness of the proposed approach. Section 4 discusses the possibilities of increasing the accuracy of the retrieval of the unknown magnetization parameters in the case of future interplanetary missions using instruments that allow measuring magnetic gradient tensor data [31,32,33].

## 2. General Problem Statement

The equation describing the magnetic field induction $B_{field}$ induced by an object with a magnetization distribution M(r) and a localization in a domain *V* has the form [34]

(1) $$B_{field}\left(r_{s}\right)=\frac{\mu_{0}}{4\pi }\underset{V}{\;\int \int \int \;}\left(\frac{3\left(M\left(r\right),r-r_{s}\right)\left(r-r_{s}\right)}{|r-r_{s}{|}^{5}}-\frac{M(r)}{|r-r_{s}{|}^{3}}\right)dv,$$

where $|r-r_{s}|=\sqrt{{\left(x-x_{s}\right)}^{2}+{\left(y-y_{s}\right)}^{2}+{\left(z-z_{s}\right)}^{2}}$ is a distance between the point $r_{s}=\left(x_{s},y_{s},z_{s}\right)$ , at which the triaxial sensor *s* measuring magnetic field induction $B_{field}$ is located, and the point r=(x,y,z) of the domain *V*, at which a magnetic source with a total magnetic moment per unit volume M(r) is placed, $\mu_{0}$ is a permeability in vacuum.

The **inverse problem** is to determine the magnetic moment density M(r) , r∈V , by using measured magnetic field induction $B_{field}\left(r_{s}\right)$ at points $r_{s}$ , $s=\bar{1,S}$ .

**Remark** **1.** *Note that this problem statement is physically defined. In the present formulation, it is necessary to restore one vector function from the results of measurements of one vector function as well. Taking into account the fact that each component of a vector function is a scalar function, it requires reconstructing three scalar functions from the results of measurements of three scalar functions. This formulation leads to a system of three scalar equations with three unknown functions.*

## 3. Reconstruction of the Equivalent Distribution of Magnetization Parameters in the Near-Surface Layer (Crust) of Mars

For convenience, we rewrite Equation (1) as

(2) $$B_{field}\left(x_{s},y_{s},z_{s}\right)=\frac{\mu_{0}}{4\pi }\underset{V}{\;\int \int \int \;}K_{TMI}\left(x_{s},y_{s},z_{s},x,y,z\right)\;M\left(x,y,z\right)\;dv,$$

where

$$\begin{array}{cc} \; & K_{TMI}\left(x_{s},y_{s},z_{s},x,y,z\right)=\\ \; & =\frac{1}{r^{5}}\left[\begin{array}{ccc} 3{\left(x-x_{s}\right)}^{2}-r^{2} & 3\left(x-x_{s}\right)\left(y-y_{s}\right) & 3\left(x-x_{s}\right)\left(z-z_{s}\right)\\ 3\left(y-y_{s}\right)\left(x-x_{s}\right) & 3{\left(y-y_{s}\right)}^{2}-r^{2} & 3\left(y-y_{s}\right)\left(z-z_{s}\right)\\ 3\left(z-z_{s}\right)\left(x-x_{s}\right) & 3\left(z-z_{s}\right)\left(y-y_{s}\right) & 3{\left(z-z_{s}\right)}^{2}-r^{2} \end{array}\right]. \end{array}$$

Here, for shorthand, we use the notation:

$$r\equiv |r-r_{s}|=\sqrt{{\left(x-x_{s}\right)}^{2}+{\left(y-y_{s}\right)}^{2}+{\left(z-z_{s}\right)}^{2}}.$$

**Figure 1:**
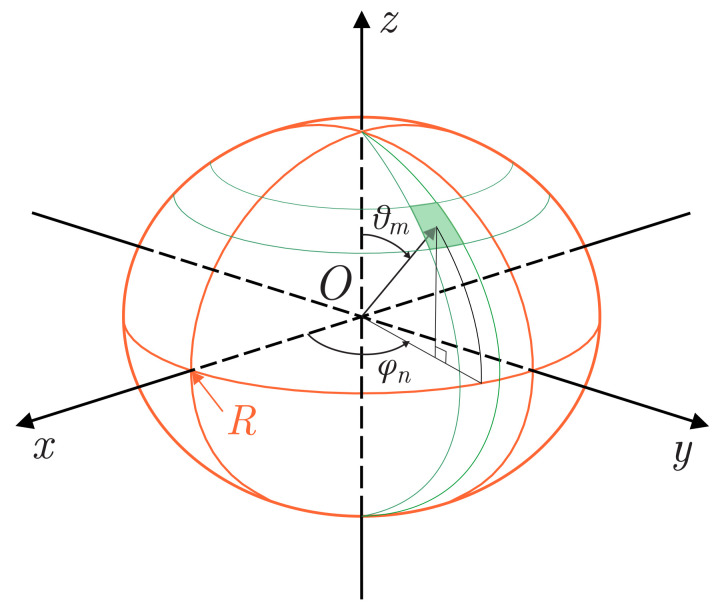
Planetary coordinate systems used to solve the problem.

In addition to the default Cartesian coordinate system, we also introduce a spherical coordinate system (see Figure 1):

(3) x=ρcosφsinθ,y=ρsinφsinθ,z=ρcosθ,ρ∈[0,R],θ∈[0,π],φ∈[0,2π).

Here, *R* is the average radius of Mars.

Based on the assumption that the region *V* is a near-surface spherical layer of Mars of depth *h* and taking into account (3), Equation (2) can be rewritten as:

(4) $$\begin{array}{cc} \; & B\left(x_{s},y_{s},z_{s}\right)=\frac{\mu_{0}}{4\pi }\int_{R-h}^{R}\int_{0}^{2\pi }\int_{0}^{\pi }K\left(x_{s},y_{s},z_{s},\rho \cos\varphi \sin\theta ,\rho \sin\varphi \sin\theta ,\rho \cos\theta \right)\cdot \\ \; & \;\cdot M\left(\rho \cos\varphi \sin\theta ,\rho \sin\varphi \sin\theta ,\rho \cos\theta \right)\cdot \rho^{2}\sin\theta \cdot d\rho \;d\phi \;d\theta . \end{array}$$

**Remark** **2.** *Here $B\equiv B_{field}$ and $K\equiv K_{TMI}$ . The introduction of additional notation is due to the fact that the matrix K (and, accordingly, the vector-function B ) can be extended by taking into account additional physical information in the formulation of the problem. This will be done at the end of Section 4.*

Let us introduce grids $\Phi_{N_{\varphi }}=\left\{\varphi_{n},1\leq n\leq N_{\varphi }:\varphi_{n}=\frac{h_{\varphi }}{2}+h_{\varphi }\;\left(n-1\right),\;h_{\varphi }=\frac{2\pi }{N_{\varphi }}\right\}$ and $\Theta_{N_{\theta }}=\left\{\theta_{m},1\leq m\leq N_{\theta }:\theta_{m}=\frac{h_{\theta }}{2}+h_{\theta }\;\left(m-1\right),\;h_{\theta }=\frac{\pi }{N_{\theta }}\right\}$ . Furthermore, for simplicity, we will proceed from the assumption that the spherical layer is thin enough. As a consequence, with respect to the variable ρ we introduce a grid with only one node $\rho_{h}=R-\frac{h}{2}$ . As a result, approximating the integrals in (4) using the midpoint rule, we obtain

$$\begin{array}{cc} \; & B\left(x_{s},y_{s},z_{s}\right)=\frac{\mu_{0}}{4\pi }\sum_{n=1}^{N_{\varphi }}\sum_{m=1}^{N_{\theta }}K\left(x_{s},y_{s},z_{s},\rho_{h}\cos\varphi_{n}\sin\theta_{m},\rho_{h}\sin\varphi_{n}\sin\theta_{m},\rho_{h}\cos\theta_{m}\right)\cdot \\ \; & \;\cdot M\left(\rho_{h}\cos\varphi_{n}\sin\theta_{m},\rho_{h}\sin\varphi_{n}\sin\theta_{m},\rho_{h}\cos\theta_{m}\right)\cdot \rho_{h}^{2}\sin\theta_{m}\cdot h\;h_{\varphi }\;h_{\theta }. \end{array}$$

We take into account that (1) measurements are made for all $s=\bar{1,S}$ , (2) B and M re vector-functions (in particular, $M=M_{x}i+M_{y}j+M_{z}k$ ). Thus, we obtain a system with 3×S equations (which correspond to the measurement of three components of the vector-function B at *S* points) with $3\times N_{\varphi }\times N_{\theta }$ unknowns (which correspond to the grid values of the three components of the vector-function M on the introduced grid $\Phi_{N_{\varphi }}\times \Theta_{N_{\theta }}$ ).

To these equations we add the following natural physical conditions.

1. Matching condition along one of the meridians. This condition means that the magnetic image must be 2π -periodic in the variable φ :
$$\begin{array}{cc} \; & M(\rho_{h}\cos\varphi_{1}\sin\theta_{m},\rho_{h}\sin\varphi_{1}\sin\theta_{m},\rho_{h}\cos\theta_{m})=\\ \; & \;=M\left(\rho_{h}\cos\varphi_{N_{\varphi }}\sin\theta_{m},\rho_{h}\sin\varphi_{N_{\varphi }}\sin\theta_{m},\rho_{h}\cos\theta_{m}\right),\;m=\bar{1,N_{\theta }}. \end{array}$$
  These conditions give additional $3\times N_{\theta }$ equations.
2. Matching condition at the South Pole. This means that all the grid values of the components of vector-function M must match when $\theta =\theta_{N_{\theta }}$ :
$$\begin{array}{cc} \; & M(\rho_{h}\cos\varphi_{n}\sin\theta_{N_{\theta }},\rho_{h}\sin\varphi_{n}\sin\theta_{N_{\theta }},\rho_{h}\cos\theta_{N_{\theta }})=\\ \; & \;=M\left(\rho_{h}\cos\varphi_{n+1}\sin\theta_{N_{\theta }},\rho_{h}\sin\varphi_{n+1}\sin\theta_{N_{\theta }},\rho_{h}\cos\theta_{N_{\theta }}\right),\;n=\bar{1,N_{\varphi }-1}. \end{array}$$
  These conditions give additional $3\times (N_{\varphi }-1)$ equations.
3. Matching condition at the North Pole.
$$\begin{array}{cc} \; & M(\rho_{h}\cos\varphi_{n}\sin\theta_{1},\rho_{h}\sin\varphi_{n}\sin\theta_{1},\rho_{h}\cos\theta_{1})=\\ \; & \;=M\left(\rho_{h}\cos\varphi_{n+1}\sin\theta_{1},\rho_{h}\sin\varphi_{n+1}\sin\theta_{1},\rho_{h}\cos\theta_{1}\right),\;n=\bar{1,N_{\varphi }-1}. \end{array}$$
  These conditions give additional $3\times (N_{\varphi }-1)$ equations.

**Remark** **3.** *Note that for the chosen method of drawing the meshes, formally $\varphi_{1}\neq \varphi_{N_{\varphi }}$ , $\theta_{N_{\theta }}\neq \frac{\pi }{2}$ and $\theta_{1}\neq 0$ . However, for $N_{\varphi }\to \infty$ and $N_{\theta }\to \infty$ : $\varphi_{1}\to \varphi_{N_{\varphi }}$ , $\theta_{N_{\theta }}\to \frac{\pi }{2}$ and $\theta_{1}\to 0$ . Therefore, the sewing conditions described above are adequate in the case of sufficiently dense grids with a sufficiently large number of intervals $N_{\varphi }$ and $N_{\theta }$ .*

Thus, taking into account additional physical conditions, we obtain a system of linear algebraic equations consisting of $3S+3N_{\theta }+6N_{\varphi }-6$ equations, each of which contains $3N_{\varphi }N_{\theta }$ unknown (the grid values of the components of the vector-function M ). Note that the resulting system for a sufficiently large *S* is overdetermined only from the mathematical point of view. From a physical point of view, the resulting system is still definite.

This system of linear algebraic equations can be written in matrix form

(5) AM=B.

Here, the vector *M* contains the grid values of three components of the unknown vector function M , the first 3S components of the vector on the right side of *B* contain the results of experimental measurements of three components of the vector function B , and subsequent components of the vector *B* are zeros.

### 3.1. Using a Regularizing Algorithm

When processing experimental data, instead of exactly known vector *B* and matrix *A* their approximate values $B_{\delta }$ and $A_{h}$ are usually known, such that $∥B_{\delta }{-B∥}_{E}⩽\delta$ , $∥A-A_{h}{∥}_{E\to E}⩽h$ . This is due both to errors in the measurements of the magnetic field (they add an error in the values of the components of the vector *B*) and to errors in the accuracy of determining the position of the satellite relative to Mars (they add an error in the values of the components of the matrix *A*). Under the described conditions, the problem is ill-posed. To solve it, it is necessary to construct a regularizing algorithm. We will use an algorithm based on minimization of the Tikhonov functional [35]

(6) $$F^{\alpha }\left[M\right]=∥A_{h}M-B_{\delta }{∥}_{E}^{2}+\alpha {∥M∥}_{E}^{2}.$$

For any α>0 there is a unique extremal of the Tikhonov functional $M_{\eta }^{\alpha }$ , η={δ,h} , realizing the minimum of $F^{\alpha }\left[M\right]$ . The algorithm of the generalized residual principle [35] can be used to select the regularization parameter. Then, choosing the parameter α=α(η) according to the generalized residual principle

$$\rho \left(\alpha \right)\equiv ∥A_{h}M_{\eta }^{\alpha }-B_{\delta }{∥}_{E}^{2}-{\left(\delta +h∥M_{\eta }^{\alpha }{∥}_{E}\right)}^{2}=0,$$

$M_{\eta }^{\alpha }$ tends to exact solution with η→0 .

The method of conjugate gradients is used as a method for minimizing the Tikhonov functional.

### 3.2. Using the Conjugate Gradient Method

Let $M^{\left(k\right)}$ be a minimizing sequence, $p^{\left(k\right)}$ and $q^{\left(k\right)}$ be auxiliary vectors, and $p^{\left(0\right)}=0$ , $M^{\left(1\right)}$ — any admissible point. Then, the formulas of the conjugate gradient method for finding the element $M^{\left(3N_{\varphi }N_{\theta }\right)}$ , realizing the minimum of the functional (6) can be written as

$$\begin{array}{cc} \; & r^{\left(k\right)}=\left\{\begin{array}{cccccc} & A_{h}^{T}\left(A_{h}\;M^{\left(k\right)}-B_{\delta }\right)+\alpha \;M^{\left(k\right)}, & \; & if & \; & k=1,\\ & r^{\left(k-1\right)}-q^{\left(k-1\right)}/\left(p^{\left(k-1\right)},q^{\left(k-1\right)}\right), & \; & if & \; & k⩾2, \end{array}\right.\\ \; & p^{\left(k\right)}=p^{\left(k-1\right)}+\frac{r^{\left(k\right)}}{\left(r^{\left(k\right)},r^{\left(k\right)}\right)},\\ \; & q^{\left(k\right)}=A_{h}^{T}\left(A_{h}\;p^{\left(k\right)}\right)+\alpha \;p^{\left(k\right)}\;,\\ \; & M^{\left(k+1\right)}=M^{\left(k\right)}-\frac{p^{\left(k\right)}}{\left(p^{\left(k\right)},q^{\left(k\right)}\right)}. \end{array}$$

**Remark** **4.** *It should be noted that during numerical experiments we define α=0 , $M^{\left(1\right)}=0$ and use the iteration number k as the regularization parameter. In this case, the criterion for terminating the iterative process is consistent with the error in specifying the input data by means of the condition [36]*

$$∥A_{h}\;M^{\left(k+1\right)}-B_{\delta }{∥}_{E}^{2}\leq {\left(\delta +h∥,M_{\eta }^{\alpha },{∥}_{E}\right)}^{2}.$$

When numerically searching for the minimum of the functional (6) it is possible to effectively use multiprocessor systems, the specifics of working with these are detailed in [37,38,39].

### 3.3. Experimental Data Processing Results

**Figure 2:**
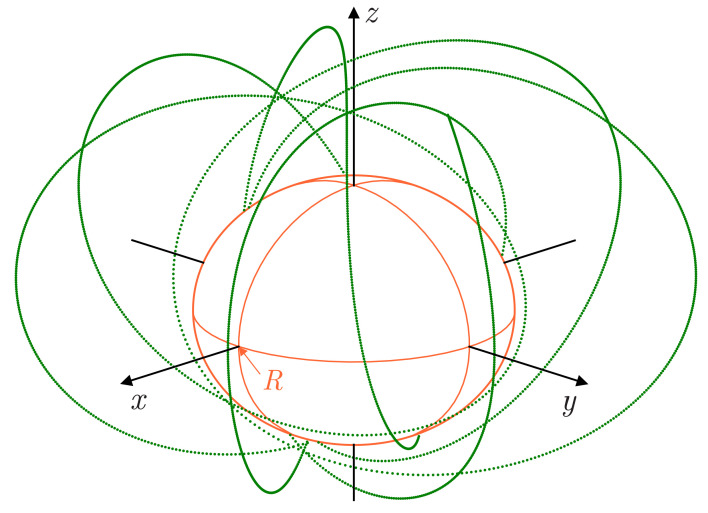
The location relative to Mars of the magnetic field measurement points by the MAVEN mission used in the calculations.

**Figure 3:**
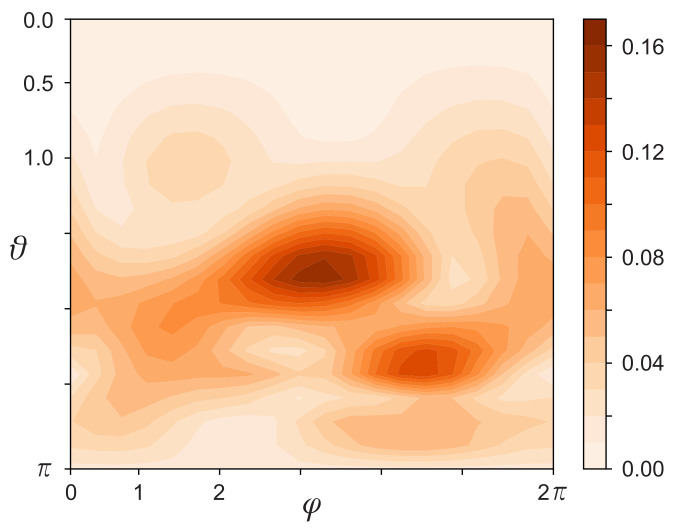
The normalized value of the magnitude of the retrieved magnetic moment density M(Rcosφsinθ,Rsinφsinθ,Rcosθ) .

The data [17] of observations of NASA’s Mars MAVEN orbiter [18] were taken as experimental data. The observation area was a set of points at which the magnetic induction of Mars was measured on the 135th day of 2020. Every 32nd point was taken from the data file [17], starting with the first and ending with the 86400th (see Figure 2). Thus, S=2700 . For calculations, grids with the number of intervals $N_{\varphi }=200$ and $N_{\theta }=200$ were used. The grid data made it possible to reconstruct the qualitative distribution of the magnetization parameters over the surface of Mars. The calculations used the values for the average radius of Mars R= 3,389,500 (m) and the thickness of the near-surface layer h=1000 (m). The results are shown in Figure 3 in polar coordinates. Note that changes in the value of *h* will lead to a proportional change in the values of the components of the vector M , but the picture of its normalized value shown in Figure 3 will remain unchanged.

## 4. Discussion

In recent years, with the development of advanced technology, acquisition of the full tensor gradient magnetic data became available. Much research has shown the advantages of magnetic gradient tensor (MGT) surveys as compared to the conventional total magnetic intensity (TMI) surveys [40,41,42,43,44]. The main conclusion is that better inversion results can be obtained with full MGT data. Thus, we see this approach as promising in the future, since the use of solely magnetic data does not always give satisfactory results. Some work notes their insufficiency and the importance of using data on the magnetic field at low altitudes, which makes it possible to increase the accuracy of retrieving the desired magnetic parameters when using the TMI model.

We define full tensor magnetic gradient $B_{tensor}$ , which unlike the magnetic induction $B_{field}$ (that has only three components) has nine components and can be written in the following matrix form [31]:

$$B_{tensor}\equiv \left[B_{ij}\right]\equiv \left[\begin{array}{ccc} \frac{\partial B_{x}}{\partial x} & \frac{\partial B_{x}}{\partial y} & \frac{\partial B_{x}}{\partial z}\\ \frac{\partial B_{y}}{\partial x} & \frac{\partial B_{y}}{\partial y} & \frac{\partial B_{y}}{\partial z}\\ \frac{\partial B_{z}}{\partial x} & \frac{\partial B_{z}}{\partial y} & \frac{\partial B_{z}}{\partial z} \end{array}\right]\equiv \left[\begin{array}{ccc} B_{xx} & B_{xy} & B_{xz}\\ B_{yx} & B_{yy} & B_{yz}\\ B_{zx} & B_{zy} & B_{zz} \end{array}\right],$$

where $B_{x}$ , $B_{y}$ and $B_{z}$ are components of the vector-function $B_{field}$ .

Note that $\frac{\partial B_{x}}{\partial y}=\frac{\partial B_{y}}{\partial x}$ , $\frac{\partial B_{x}}{\partial z}=\frac{\partial B_{z}}{\partial x}$ , $\frac{\partial B_{y}}{\partial z}=\frac{\partial B_{z}}{\partial y}$ and $\frac{\partial B_{x}}{\partial x}+\frac{\partial B_{y}}{\partial y}+\frac{\partial B_{z}}{\partial z}=0$ . So, actually, we have only five different components of the tensor matrix.

The diagonal elements and non-diagonal elements of tensor matrix $B_{tensor}$ have the form (details of the derivation of these formulas are presented in [31]):

$$\begin{array}{cc} \; & B_{ii}=\frac{\mu_{0}}{4\pi }\underset{V}{\;\int \int \int \;}\left(\frac{6M_{i}\left(r\right)\left(i-i_{s}\right)}{r^{5}}+\frac{3\left(M\left(r\right),r-r_{s}\right)}{r^{5}}-\frac{15\left(M\left(r\right),r-r_{s}\right)\left(i-i_{s}\right)\left(i-i_{s}\right)}{r^{7}}\right)dv,\\ \; & B_{ij}=\frac{\mu_{0}}{4\pi }\underset{V}{\;\int \int \int \;}\left(\frac{3M_{i}\left(r\right)\left(j-j_{s}\right)}{r^{5}}+\frac{3M_{j}\left(r\right)\left(i-i_{s}\right)}{r^{5}}-\frac{15\left(M\left(r\right),r-r_{s}\right)\left(i-i_{s}\right)\left(j-j_{s}\right)}{r^{7}}\right)dv. \end{array}$$

Here, for each character from the set {i,j} one of the characters must be substituted among {x,y,z} .

By adding these equations to Equation (2), we obtain the following system of two 3D Fredholm integral equations of the 1st kind:

(7) $$\left\{\begin{array}{cc} \; & B_{field}\left(x_{s},y_{s},z_{s}\right)=\frac{\mu_{0}}{4\pi }\underset{V}{\;\int \int \int \;}K_{TMI}\left(x_{s},y_{s},z_{s},x,y,z\right)\;M\left(x,y,z\right)\;dv,\\ \; & B_{tensor}\left(x_{s},y_{s},z_{s}\right)=\frac{\mu_{0}}{4\pi }\underset{V}{\;\int \int \int \;}K_{MGT}\left(x_{s},y_{s},z_{s},x,y,z\right)\;M\left(x,y,z\right)\;dv, \end{array}\right.$$

where $B_{field}={\left[B_{x}\;B_{y}\;B_{z}\right]}^{T}$ and $B_{tensor}={\left[B_{xx}\;B_{xy}\;B_{xz}\;B_{yz}\;B_{zz}\right]}^{T}$ . Kernel $K_{MGT}$ of the second integral equation can be written as

$$\begin{array}{cc} \; & K_{MGT}\left(x_{s},y_{s},z_{s},x,y,z\right)=\\ \; & \;=\frac{3}{r^{7}}\;\left[\begin{array}{ccc} \left(x-x_{s}\right)\left[3r^{2}-5{\left(x-x_{s}\right)}^{2}\right] & \;\left(y-y_{s}\right)\left[r^{2}-5{\left(x-x_{s}\right)}^{2}\right] & \;\left(z-z_{s}\right)\left[r^{2}-5{\left(x-x_{s}\right)}^{2}\right]\\ \left(y-y_{s}\right)\left[r^{2}-5{\left(x-x_{s}\right)}^{2}\right] & \;\left(x-x_{s}\right)\left[r^{2}-5{\left(y-y_{s}\right)}^{2}\right] & \;-5\left(x-x_{s}\right)\left(y-y_{s}\right)\left(z-z_{s}\right)\\ \left(z-z_{s}\right)\left[r^{2}-5{\left(x-x_{s}\right)}^{2}\right] & \;-5\left(x-x_{s}\right)\left(y-y_{s}\right)\left(z-z_{s}\right) & \;\left(x-x_{s}\right)\left[r^{2}-5{\left(z-z_{s}\right)}^{2}\right]\\ -5\left(x-x_{s}\right)\left(y-y_{s}\right)\left(z-z_{s}\right) & \;\left(z-z_{s}\right)\left[r^{2}-5{\left(y-y_{s}\right)}^{2}\right] & \;\left(y-y_{s}\right)\left[r^{2}-5{\left(z-z_{s}\right)}^{2}\right]\\ \left(x-x_{s}\right)\left[r^{2}-5{\left(z-z_{s}\right)}^{2}\right] & \;\left(y-y_{s}\right)\left[r^{2}-5{\left(z-z_{s}\right)}^{2}\right] & \;\left(z-z_{s}\right)\left[3r^{2}-5{\left(z-z_{s}\right)}^{2}\right] \end{array}\right]. \end{array}$$

Thus, using the full magnetic gradient tensor in Equation (4) the structure of B and K will change. Now $B\equiv {\left[B_{field}\;B_{tenzor}\right]}^{T}$ and $K\equiv {\left[K_{TMI}\;K_{MGT}\right]}^{T}$ .

As a result, we obtain a physically overdetermined problem, which consists of determining three unknown scalar functions from the results of experimental measurements of the other eight scalar functions. The accuracy of recovering the unknown functions from this formulation of the inverse problem will be much higher [31].

## 5. Conclusions

The method developed by the authors for determining the magnetic properties of rocks from satellite data can be successfully applied in the process of complex interpretation of data (the so-called joint inversion) on the physical fields of Mars and its topography. The distribution of the magnetization parameters equivalent to the external field, found using the algorithm presented in the article, makes it possible to recreate a qualitative picture of the internal structure of the planet [45,46,47,48,49]. This is due to the fact that the magnetic permeability and magnetic susceptibility are closely related to such magnetic distributions. In the future, it is planned to test the methodology when solving problems in various statements, both simpler and more complicated. It is very important to emphasize that we are processing a dataset all over Mars, i.e., we solve the problem of interpretation in the global version. The method described in the article provides a high quality interpretation of the data on the magnetic field of Mars, which were obtained from satellites flying at different heights above the surface of the Red Planet. To take into account the nuances of mathematical formulations, a more detailed study of the local features of the magnetic field of the Red Planet is required. We hope that, as information comes from the surface of the planet, we will be able to improve the accuracy and reliability of our method. Building an analytical model of the magnetic field of Mars from satellite data is a difficult problem that researchers from different countries are trying to solve. Verification of certain theoretical studies can be carried out only on the basis of experimental measurement data. At the same time, the difficulties that theorists face when solving interpretation problems can serve as an incentive for technologists and developers of space equipment when planning an experiment.

## Data Availability

Not applicable.
